# The Genome-Wide EMS Mutagenesis Bias Correlates With Sequence Context and Chromatin Structure in Rice

**DOI:** 10.3389/fpls.2021.579675

**Published:** 2021-03-24

**Authors:** Wei Yan, Xing Wang Deng, Chengwei Yang, Xiaoyan Tang

**Affiliations:** ^1^Guangdong Provincial Key Laboratory of Biotechnology for Plant Development, School of Life Sciences, South China Normal University, Guangzhou, China; ^2^Shenzhen Institute of Molecular Crop Design, Shenzhen, China; ^3^School of Life Sciences, Southern University of Science and Technology, Shenzhen, China

**Keywords:** EMS mutagenesis bias, EMS-induced SNPs, natural SNPs, sequence context, chromatin structure, DNA repair

## Abstract

Ethyl methanesulfonate (EMS) is a chemical mutagen believed to mainly induce G/C to A/T transitions randomly in plant genomes. However, mutant screening for phenotypes often gets multiple alleles for one gene but no mutant for other genes. We investigated the potential EMS mutagenesis bias and the possible correlations with sequence context and chromatin structure using the whole genome resequencing data collected from 52 rice EMS mutants. We defined the EMS-induced single nucleotide polymorphic sites (SNPs) and explored the genomic factors associated with EMS mutagenesis bias. Compared with natural SNPs presented in the Rice3K project, EMS showed a preference on G/C sites with flanking sequences also higher in GC contents. The composition of local dinucleotides and trinucleotides was also associated with the efficiency of EMS mutagenesis. The biased distribution of EMS-induced SNPs was positively correlated with CpG numbers, transposable element contents, and repressive epigenetic markers but negatively with gene expression, the euchromatin marker DNase I hypersensitive sites, and active epigenetic markers, suggesting that sequence context and chromatin structure might correlate with the efficiency of EMS mutagenesis. Exploring the genome-wide features of EMS mutagenesis and correlations with epigenetic modifications will help in the understanding of DNA repair mechanism.

## Introduction

Mutation breeding is an efficient way to obtain desirable traits in crop plants (Pathirana, 2011; Sikora et al., 2011). The first record of using a spontaneous mutant can be traced back to 300 BC in ancient China in cereal crops (Shu and Forster, 2011). However, the spontaneous mutation rate is only about 10^–8^–10^–7^ events per base pair per generation (Kovalchuk et al., 2000), making it extremely inefficient to obtain a desirable trait. Since the start of mutation breeding programs in the 1930s, mutants with desirable agronomic traits or elimination of undesirable traits induced by radiation, chemical mutagens, or transposon insertion have been widely discovered in multiple plant species (Koornneef et al., 1982; Pathirana, 2011; Hola et al., 2015). More than 3,320 mutant varieties in 242 species, including numerous crops, trees, and ornamentals with improved yield, quality, adaptation, or resistance to diseases, have been released for commercial use worldwide, according to the registration in FAO/IAEA Mutant Variety Database (MVD)^1^. With the development of various mutagenesis technologies and tools to explore genomic changes, many databases have been established for the collection of induced mutant plants of various species (Beale et al., 2002; Zhang et al., 2006; Saito et al., 2011; Li et al., 2017; Lu et al., 2017). These databases play a significant role in advancing the research and development of plant science and crop breeding. Among the mutants collected in MVD that were induced by chemical mutagens, more than 80% of them were induced by alkylation agents.

Generally, the alkylation agents cause mutations by introducing alkyl groups at specific sites of the nucleotides, including the *N*^7^ and *O*^6^ of guanine (G), *N*^3^ and *N*^7^ of adenine (A), *N*^3^, *O*^2^, and *O*^4^ of thymine (T), *N*^3^, *O*^2^, and *N*^4^ of cytosine (C), and the phosphonate backbone (Leitao, 2011; Xu et al., 2017). The *O*^2^-, *N*^3^-, and *O*^4^-ethylthymidine lesions can be recognized and bypassed by RNA polymerase II (pol II) (Xu et al., 2017). *N*^7^-alkylguanine is still recognized as guanine during DNA mismatch repair, i.e., this alkylation is non-mutagenic. Guanine with alkylation at *O*^6^-guanine can pair up with thymine but not cytosine, resulting in the replacement of the G/C pair with A/T in subsequent DNA repair (Greene et al., 2003). In prokaryotes and mammals, the alkylation at *O*^6^-guanine can be partially repaired by DNA alkyltransferases through a direct reversal and suicidal mechanism to transfer the alkyl group to a cysteine of the active site (Drablos et al., 2004; Shrivastav et al., 2010; Fu et al., 2012). However, there are no homologs of DNA alkyltransferases identified in plants; therefore, alkylation agents mainly induce G/C to A/T transitions in plants (Koornneef et al., 1982; Kim et al., 2006).

Ethyl methanesulfonate (EMS) (C_3_H_8_O_3_S) is one of the widely used chemical alkylation agents that mainly introduces an alkyl group at *N*^7^-guanine and *O*^6^-guanine. Previous studies showed that more than 99% EMS-induced variations in 192 targeted genes were G/C to A/T transitions in *Arabidopsis* (Greene et al., 2003), whereas these two types of transitions occupied only 79.8% and 70% in EMS-induced mutations in maize and rice populations, respectively (Till et al., 2007; Lu et al., 2017). The varied percentages of G/C to A/T transitions induced by EMS in different species may be related to the genetic compositions and/or genomic features of these organisms.

Similar to the mutations induced by radiation, EMS was believed to induce random mutations genome-wide regardless of chromatin structures or DNA sequences (Greene et al., 2003). However, by screening the 3,072 EMS-mutagenized M_2_ plants with TILLING in *Arabidopsis*, some of the 192 targeted fragments were indeed found to harbor more allelic mutations than average (Greene et al., 2003). Additionally, local compositional biases were also detected for the nearest two neighbor bases on either side of the mutated guanine, i.e., purines were overrepresented at −1 and +1, and guanines were deficient at −2 but excessive at +2 positions (Greene et al., 2003). During the identification of causal genes for mutants of different phenotypes from an EMS-induced *indica* rice variety Huanghuazhan (HHZ) mutant library with SIMM (Yan et al., 2017), we also detected multiple allelic mutants for some genes while there was no mutation for other genes controlling the same phenotype, indicating that the mutations induced by EMS might not be randomly distributed. Although EMS has been widely applied in plant mutation breeding, the genome-wide features of EMS mutagenesis and the potential factors affecting EMS mutagenesis are still unclear.

Here we collected the bulk-sequencing data of 52 HHZ mutants and integratively analyzed with the flanking DNA sequence composition, gene expression data, chromatin structure, and chromatin modification data of rice to evaluate the potential EMS mutagenesis bias and to explore genomic factors affecting the genome-wide bias of EMS mutagenesis. We found that EMS-induced mutations were not uniformly distributed in chromosome and preferred regions with higher GC contents, higher CpG numbers, more transposable elements, low levels of gene expression, and heterochromatin features. Through the integrative analyses of public chromatin modification data, we also found that epigenetic modifications, especially DNA methylation and histone acetylation, might also correlate with the genome-wide bias of EMS mutagenesis.

## Materials and Methods

### Selection of EMS-Induced Mutants for Whole-Genome Re-sequencing Analysis

An EMS mutant library was constructed previously using seeds from the *indica* variety HHZ (Yan et al., 2017). Briefly, 50 kg of HHZ seeds were pre-soaked in water for 22 h and then treated with 0.7% EMS solution for 12 h at 28°C. The germinated seeds were planted in the field, and the plants were divided into 600 pools, and seeds from each pool were mix-harvested to form the EMS mutant library. Mutants with segregated phenotypes in F_2_ populations were selected for further study. A total of 52 recessive mutants from this library with stable phenotypes in fertility, plant architecture, grain size, panicle numbers, or leaf morphology were selected for re-sequencing analysis in this study. Firstly, each mutant was backcrossed with the wild-type progenitor and then selfed to obtain the F_2_ segregating population. Then, 30 individuals with the mutant phenotype from each F_2_ population were randomly selected, equally pooled, and re-sequenced on Illumina Hiseq platforms (PE150/250 with insert size that ranged from 300 to 500 bp), resulting in 52 sequenced bulks in total.

### Identification of Genome-Wide EMS-Induced SNPs

The re-sequencing data (∼39× of the genome size on average) of each mutant bulk were aligned to the Nipponbare reference genome (MSU v7.0) after quality control to identify the total SNPs for each mutant bulk, including the natural SNPs between HHZ and Nipponbare as well as EMS-induced SNPs. The resulting total SNPs for each of the 52 bulks were then compared among each other using the SIMM method to exclude the natural SNPs common among the mutants (Yan et al., 2017). SNP index (SI) and Euclidean distance (ED) were used to evaluate each putative EMS-induced SNP in the bulked sequences. SI indicates the ratio of the mutant reads to the total reads in the test mutant bulk, and theoretically, SI is expected to be 1 for the causal mutation site and around 0.5 for other SNPs unlinked to the causal mutation. ED was raised to the power of 6 to enlarge the differences between the test mutant bulk and other mutant bulks. ED^6^ indicates the linkage between SNPs and the mutant phenotype, and SNPs closely linked to the mutant phenotype have a bigger ED^6^ value (Yan et al., 2017). Because the Illumina Hiseq platform can generate random sequencing errors, we allowed 20% sequencing errors in the SIMM algorithm. Only SNPs supported by at least three mutant reads with mapping quality ≥20 in the test mutant bulk and the average mutant reads in other mutant bulks ≤1 were retained as reliable EMS-induced SNPs. Some mutants harbor fragments with a very high density of SNPs. These fragments were likely the residual fragments in heterozygosity inherited from the progenitor parents for breeding of HHZ, and SNPs in these fragments were not induced by EMS and thus were excluded from further analysis. Only SNPs presented in one mutant and completely absent in all other 51 mutants were retained as EMS-induced mutations.

To illustrate the genomic features associated with EMS-induced SNPs, the identified EMS-induced SNPs were compared with the natural SNPs generated by the Rice3K project, in which 3,010 rice germplasms were re-sequenced to uncover the within-species diversity and genome-level population structures of *Oryza sativa* (Li et al., 2014). Only biallelic SNPs presented in the Rice3K project and supported by at least 30 varieties with minor allele frequency (MAF) ≥0.01 and missing rate <0.8 were retained as reliable natural SNPs for comparative analysis. Missing rate represents the percentage of cultivars lacking genotyping information among the 3,010 rice germplasms at each SNP. Paired *t*-test was used to estimate the differences of mutation types between EMS-induced SNPs and natural SNPs.

### Statistics of Base Contents and Codon Usages

The 50-bp sequences on both sides of the EMS-induced SNPs and natural SNPs were extracted from the Nipponbare reference genome as arbitrary values for calculating base contents. The average GC contents were calculated for the mutation sites, the flanking 1-bp and the flanking 50-bp sequences, respectively. All types of dinucleotides and trinucleotides around the EMS-induced SNPs were extracted, and the number for each type was counted separately for calculation of their percentages of the total. The same analyses were also performed on the natural SNPs in the Rice3K project and randomly selected sites (∼10,000 sites, repeated 10 times). The percentages of dinucleotides and trinucleotides were then compared between the EMS-induced SNPs and natural SNPs to evaluate the impact of nearby nucleotides on EMS mutagenesis. Chi-square test was used to estimate the differences of enriched dinucleotides and trinucleotides between EMS-induced SNPs and natural SNPs.

For non-synonymous (NS) SNPs induced by EMS, the number for each codon and the corresponding original amino acid and substitution amino acid were counted for the calculation of their ratio to the total amino acids. The percentage of each codon and corresponding amino acid in the Nipponbare reference genome was calculated based on the annotation of genes. Only the longest protein sequence of each gene was used for calculation.

### Characterization of “Hotspots” and “Coldspots” of EMS-Induced SNPs

Because the whole genome sequence of HHZ was not assembled, we relied on the Nipponbare genome sequence as a reference in our analyses. The Nipponbare genome was divided into 100-kb overlapped bins with a step size of 50 kb for all statistical analyses. We also chose 20 and 25 kb as step size to estimate the distribution patterns of EMS-induced SNPs along chromosomes. The number of SNPs in each bin was calculated for EMS-induced SNPs. Considering the average distance between adjacent EMS-induced SNPs and the Chi-square test results (*p*-value < 0.05), bins with at least 10 EMS-induced SNPs were designated as “hotspots,” while bins without EMS-induced SNPs were designated as “coldspots.”

### Distribution of CpG Islands and TEs Around SNPs

Sequences with more than 50% CG are usually defined as CpG islands (McWilliam et al., 2013). Genome-wide CpG islands were firstly predicted with the Nipponbare reference genome using Newcpgreport with default parameters (length ≥200 bp, window size = 100 bp, observed/expected ≥0.6, GC% ≥50%) (McWilliam et al., 2013). Then, the CpG islands within each 100-kb bin were counted and compared in EMS “hotspots” and “coldspots” as well as bins harboring 0–5 and 5–10 EMS-induced SNPs. Moreover, 5-kb sequences on both sides of the EMS-induced SNPs were also extracted for prediction of CpG islands. The SNPs were then separated into different groups according to the various CpG numbers in the 5-kb flanking sequences. The percentage of EMS-induced SNPs in each of these groups was calculated by dividing the SNP number in the particular group by the total number of EMS-induced SNPs. The same procedures were also applied to the analysis of natural SNPs. To further evaluate the correlations between EMS-induced SNPs and CpG islands, we also randomly extracted ∼10,000 10-kb sequences genome-wide to predict the CpG islands and repeated this procedure for 10 times. The distribution of transposable element (TE) genes and non-TE genes in each bin was determined according to the functional annotation of the Nipponbare reference genome (MSU v7.0).

### Chromatin Epigenetic Modification Data Analysis

DNase I hypersensitive (DH) sites are symbols for regulatory elements that can be used to represent the structure of chromatin (Zhang et al., 2012). To elucidate the correlation between EMS-induced SNPs and chromatin structure, rice DH sites identified for seedlings and callus of Nipponbare were downloaded from the NCBI Gene Expression Omnibus^2^ with accession number GSE26610 (Zhang et al., 2012). Sequences of DH sites with length larger than 36 bp (the length of single-ended reads) were extracted based on the TIGR release 5 Nipponbare genome and re-aligned to the Nipponbare (MSU v7.0) reference genome with HISAT2 (–no-spliced-alignment) (Kim et al., 2015). Then, the uniquely mapped DH sites from both tissues were pooled together with manual Perl scripts. DH sites located within 36 bp of adjacent DH sites were merged.

The chromatin structure can also be affected by DNA methylation and histone modifications (Liu et al., 2018). To elucidate the general correlations between EMS-induced SNPs and these chromatin structure-related modifications, the rice epigenetic modification data of Nipponbare from multiple tissues and experiments were downloaded from the Plant Chromatin State Database (PCSD) (Zhang et al., 2012; Liu et al., 2018), including nine experiments for DNA methylation, 12 experiments for histone acetylation, 26 experiments for histone methylation, seven experiments for histone variants H2A.Z, and 27 experiments related to chromatin status. The processed bigWig files of each epigenetic modification were transformed to signals with bwtool (Pohl and Beato, 2014). The flanking 150 bp of each modified locus with signal was extracted based on the TIGR6.1 reference genome and realigned to the Nipponbare reference genome (MSU v7.0) with SOAP2 (Li et al., 2009) to get the corresponding positions. Only those modified loci with flanking sequences that were uniquely mapped were retained. The epigenetic modifications at each covered SNP were represented with the maximum signal of different experiments. For SNPs not covered, the maximum signal of the flanking 10 bp was used for each epigenetic modification. The average signals were then calculated with the maximum signals of SNPs located in bins harboring 0–5, 5–10, and ≥10 EMS-induced SNPs, respectively. Only modifications with signals covering at least half of the EMS-induced SNPs were retained for our analysis.

## Results

### Genome-Wide Identification of EMS-Induced SNPs in HHZ Mutants

We previously isolated a large number of recessive mutants of various phenotypes from an EMS mutant library derived from the *indica* rice variety HHZ. The mutant was backcrossed with the wild-type HHZ plant, and 30 F_2_ individuals of the mutant phenotype were bulk-sequenced. The re-sequencing data of the 52 bulks were individually aligned to the Nipponbare reference genome (MSU v7.0), and the resulting SNPs for the 52 bulks were then compared among each other using the SIMM method to identify EMS-induced SNPs as described in “Materials and Methods” (Yan et al., 2017). The analysis identified a total of 17,397 EMS-induced SNPs in the 52 HHZ mutants (Supplementary Table 1). A comparison of these SNPs with the re-sequencing data of a wild-type HHZ plant verified the mutations. As expected, most of the SNPs have a SNP index that ranged between 0.4 and 0.6 (Supplementary Figure 1 and Supplementary Table 1). The number of EMS-induced SNPs in each mutant ranged from 80 to 1,091, with an average of 334.55 SNPs per mutant (Supplementary Table 2 and Supplementary Figure 1). The average distance between two adjacent SNPs ranged from 332.89 kb to 3.19 Mb in each individual mutant (Supplementary Figure 2A). When all the 17,397 SNPs were fixed on rice genome, the average distance between two adjacent SNPs was 21.43 kb (Supplementary Figure 2B). Additionally, 8,011 of these SNPs (46.05%) were separated from each other by an adjacent distance <10 kb (Supplementary Figure 2B), implying the existence of mutation-rich regions as well as mutation-rare regions in the genome. When estimating the distributions of these SNPs with a 100-kb window size, we found that ∼96.71% of 100-kb bins harbored at least one SNP, indicating that these SNPs were sufficient for analyzing the features of EMS mutagenesis in rice genome.

During the long-term evolution and breeding of rice, the rice genome has gone through innumerous natural or artificial selections, resulting in numerous variations genome-wide. The Rice3K project sequenced 3,010 rice accessions collected around the world and identified 18.9 million SNPs genome-wide using the GATK pipeline (The 3K RGP, 2014). These natural SNPs represent the diversity of rice genomes and provide a good background reference for comparative analysis of general features associated with EMS-induced SNPs. To determine whether these SNPs can be used in our study, we first downloaded the re-sequencing data of several rice lines in the Rice3K project and analyzed them with the SOAP2 pipeline that we used for the identification of EMS-induced SNPs. We found that the SNPs identified by the two pipelines were more than 93.64% identical in all the analyzed rice lines, and this was also consistent with the previous report that the GATK and SOAP2 pipelines were similar in sensitivity and specificity on SNP calling on the same dataset (O’Rawe et al., 2013). To exclude the very rare SNPs that might be derived from sequencing errors, these 18.9 million natural SNPs were firstly filtered as described in “Materials and Methods,” retaining 11,220,550 SNPs supported by at least 30 varieties with MAF ≥ 0.01 and missing rate <0.8 for comparative analysis.

### Characteristics of EMS-Induced SNPs and Nearby Flanking Sequences

**FIGURE 1:**
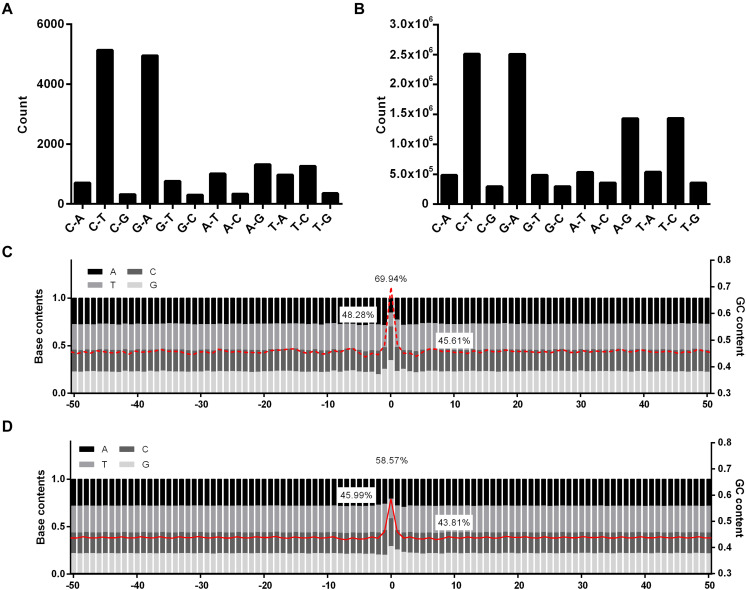
Statistics of mutation types and GC contents for ethyl methanesulfonate (EMS)-induced single nucleotide polymorphic sites (SNPs) and natural SNPs in the Rice3K project. **(A)** Mutation types of EMS-induced SNPs. **(B)** Mutation types of natural SNPs in the Rice3K project. The *y*-axis in **(A,B)** indicate the count number of each mutation type. **(C)** Base content and GC content around EMS-induced SNPs. **(D)** Base content and GC content around natural SNPs. Red lines, the average GC contents of flanking sequences. The GC contents of the mutation sites (up), +1/–1 positions (left), and flanking 50-bp sequences (right) are marked in **(C,D)**.

Of the 17,397 EMS-induced SNPs, the two well-known types of EMS-induced transitions were much enriched, occupying 29.54% (C→T) and 28.47% (G→A), respectively, followed by A→G (7.53%), T→C (7.25%), A→T (5.78%), and T→A (5.59%) (Figure 1A). Unlike the EMS-induced SNPs, the A/T to G/C substitutions were also enriched in natural SNPs besides the G/C to A/T transitions (Figure 1B). The percentage of SNPs that occurred at the G/C sites was much higher in EMS-induced SNPs (69.94%) than the natural SNPs (58.57%) presented in the Rice3K project (Figures 1C,D). The mutation types were significantly different between EMS-induced SNPs and natural SNPs (*p*-value = 0.002551).

**FIGURE 2:**
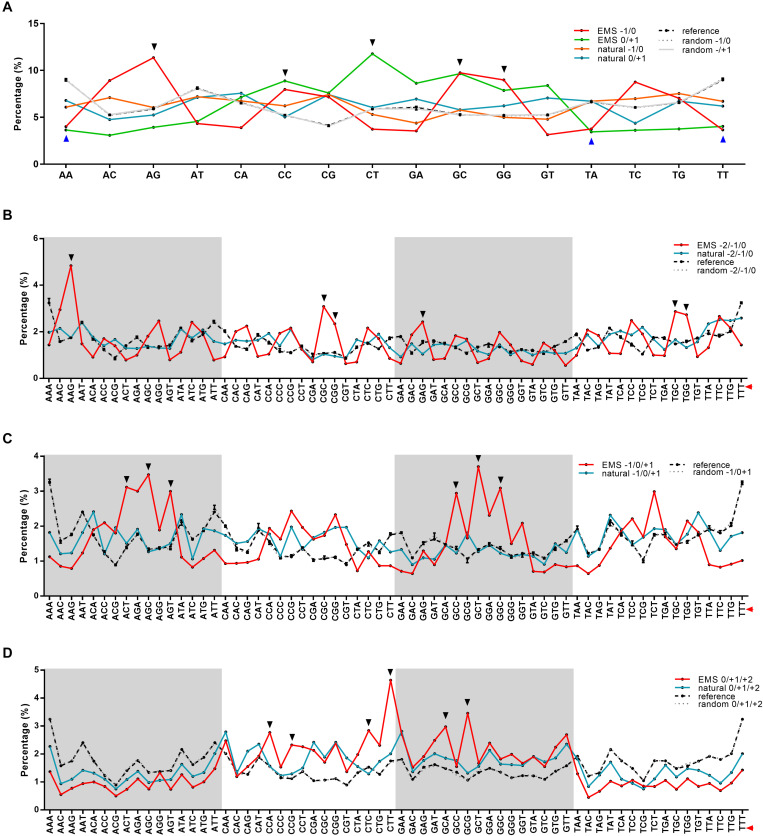
Percentages of dinucleotides and trinucleotides around the mutation sites in ethyl methanesulfonate (EMS)-induced single nucleotide polymorphic sites (SNPs) and natural SNPs in the Rice3K project. **(A)** Percentages of dinucleotides at positions –1/0 and 0/+1. **(B)** Percentages of trinucleotides at positions –2/–1/0. **(C)** Percentages of trinucleotides at positions –1/0/+1. **(D)** Percentages of trinucleotides at positions 0/+1/+2. The mutation sites are set as 0, upstream sites are indicated by –, and downstream sites are indicated by +. The percentages of each kind of dinucleotides and trinucleotides around the mutation sites are calculated and compared with their frequencies in the reference genome (dashed black lines) and randomly selected ∼10,000 sites (repeated 10 times, dashed gray lines with error bars). The mutation sites are marked with red triangles on the right for trinucleotides. Dinucleotides and trinucleotides with higher or lower frequencies in EMS-induced SNPs are marked with black triangles at the top or blue triangles at the bottom, respectively.

For nucleotides at +1/−1 positions, 48.28% adjacent to the EMS-induced SNPs (the SNP site was designated as 0) were G/C, while 45.99% adjacent to the natural SNPs were G/C (Figures 1C,D). When investigating if certain types of dinucleotides were preferred, we found five types of dinucleotides (CC, CT, and GC at positions 0/+1, AG, GG, and GC at positions −1/0) showing higher percentages in EMS-induced SNPs than natural SNPs (Figure 2A), and the difference was significant for AG and GC at positions −1/0 and CT at 0/+1 (Chi-square test *p*-value < 0.05) (Supplementary Figures 3A,B). The top two enriched dinucleotides in EMS-induced SNPs were AG (11.36%) at positions −1/0 and CT (11.77%) at positions 0/+1 (Figure 2A), suggesting a favorable local context for EMS mutagenesis. We further extended the analysis to nucleotides at +2 and −2 positions and examined if certain types of trinucleotides were preferred for EMS mutagenesis. Eighteen types of trinucleotides (AAG, CGC, CGG, GAG, TGC, and TGG at positions −2/−1/0, ACT, AGC, AGT, GCC, GCT, and GGC at positions −1/0/+1, CCA, CCG, CTC, CTT, GCA, and GCG at positions 0/+1/+2) showed higher percentages at the EMS-induced mutation sites than the natural SNPs (Figures 2B–D), and the difference was significant for AAG at positions −2/−1/0, GCT at positions −1/0/+1, and CTT and GCG at positions 0/+1/+2 (Chi-square test *p*-value < 0.05) (Supplementary Figures 3C–E). AAG (4.84%) at positions −2/−1/0 and CTT (4.64%) at positions 0/+1/+2 were the top two enriched trinucleotides in EMS-induced SNPs (Figures 2B–D). The enrichment of these dinucleotides and trinucleotides in EMS mutagenesis was neither associated with their contents in the reference genome nor correlated with their percentages in the natural SNPs (Figure 2 and Supplementary Figure 3), suggesting that these dinucleotides and trinucleotides constitute favorable local contexts for EMS mutagenesis. We also randomly selected ∼10,000 sites (repeated 10 times) and calculated the contents of dinucleotides and trinucleotides based on the Nipponbare reference genome. As expected, the contents of each dinucleotide and trinucleotide were very similar to their contents in the whole reference genome (Figure 2). These results suggested that the composition of two immediate neighbor bases might be associated with the EMS mutagenesis frequency in rice.

We further extended our analysis to 50-bp sequences flanking the SNPs as an arbitrary selection. It was found that the GC content was higher in the 50-bp sequences flanking EMS-induced SNPs (45.61%) than those flanking natural SNPs (43.81%) (Figures 1C,D). We also randomly selected ∼10,000 single nucleotide sites (repeated 10 times) from the reference genome and calculated the GC contents in the flanking 50-bp sequences, resulting in 43.54% GC on average (Supplementary Figure 4). The GC content differences in the flanking 50-bp sequences were statistically significantly different (Student’s *t*-test *p*-value = 4.42 × 10^–52^). These results indicated that EMS mutagenesis prefers the flanking sequences with relatively higher GC contents.

### Genome-Wide Effects of EMS-Induced SNPs on Genes

**FIGURE 3:**
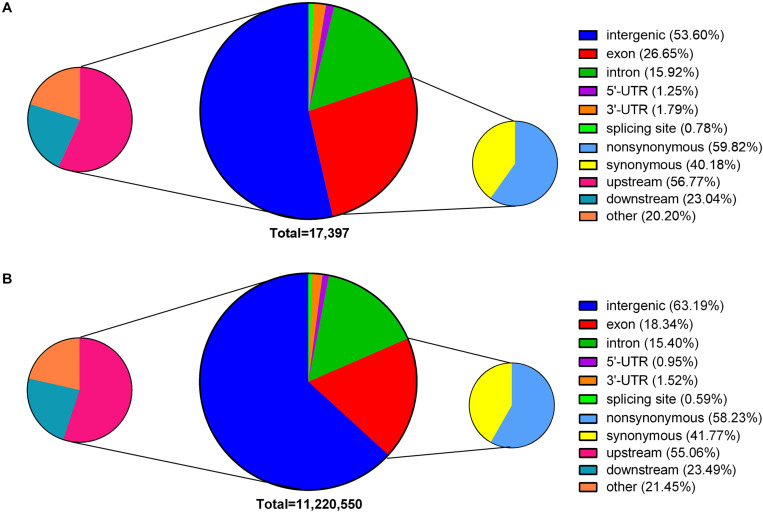
Genome-wide distribution of ethyl methanesulfonate (EMS)-induced single nucleotide polymorphic sites (SNPs) and natural SNPs. **(A)** EMS-induced SNPs. **(B)** Natural SNPs presented in the Rice3K project. SNPs within 3 kb upstream or downstream of a gene are classified as upstream and downstream. SNPs in exons are grouped into non-synonymous and synonymous according to their effects on amino acids.

According to the annotations of the Nipponbare reference genome, 43.36% of the EMS-induced SNPs were located in the gene body (26.65% in exons and 15.92% in introns) (Figure 3A), affecting 2,883 TE genes and 2,943 non-TE genes in total. Of the remaining 56.64% SNPs that were located in intergenic regions, 56.77% were within 3 kb upstream of the coding regions. In total, the identified EMS-induced SNPs could affect 4,726 TE genes and 6,706 non-TE genes, providing a large supply of mutations for reverse genetic studies. Among these affected genes, almost all the TE genes (97.04%) and 41.20% non-TE genes were expressed with an average fragments per kilobase of transcript per million mapped reads (FPKM) <1 across all the 284 RNA-seq datasets collected from the Information Commons for Rice (IC4R) database (The IC4R Project Consortium, 2016), suggesting that EMS-targeted genes tended to be inert in transcription. In addition, 59.81% of EMS-induced SNPs in exons caused amino acid substitutions, leading to NS mutations in a total of 2,385 genes. Among these genes, 302 harbored at least two NS mutant alleles. On the contrary, genome-wide distributions of natural SNPs in the Rice3K project showed a larger proportion located in intergenic regions (63.19%) and less in exons (18.34%) (Figure 3B). The presence of more natural SNPs in intergenic regions was consistent with the fact that mutations in intergenic regions have less impact on gene function and thus are allowed by natural selection.

The allele frequency in natural populations can be affected by genetic drift, natural selection, and gene flow in long-term domestication (Chen et al., 2019). Alleles with decreased frequency are often not preferred in the natural populations due to their negative effects. Interestingly, 46.38% of the EMS-induced SNPs that are shared with the Rice3K have a minor allele frequency less than 0.10 among the 3,010 rice accessions (Supplementary Table 3), suggesting that most of the EMS-induced SNPs might be harmful and could not survive the natural selection.

### Codon Usage Bias in EMS-Induced SNPs

Differential preference on local sequences implicated that EMS mutagenesis has codon usage bias. To further investigate this aspect, we calculated the frequencies of various types of amino acids induced by NS mutations before and after EMS mutagenesis. As shown in Supplementary Figures 5A,B, EMS targeted different codons and amino acids at different frequencies. In general, amino acids with low contents in the genome and low codon numbers are less targeted by EMS, while amino acids with high contents in the genome and high codon numbers are more targeted. However, we found that codons with higher GC contents were more affected by EMS (Supplementary Figure 5A). Additionally, amino acids with the same codon numbers and similar contents were not necessarily targeted by EMS at the same rates. For example, both alanine (ala) and leucine (leu) have four codons and similar contents in the genome, but 14.05% of EMS-induced mutations occurred to ala, while only 5.61% occurred to leu (Supplementary Figure 5B). Similarly, both aspartic acid (asp) and lysine (lys) have two codons and similar contents in the genome, but asp was much more preferred than lys by EMS mutagenesis (Supplementary Figure 5B). Furthermore, both arginine (arg) and serine (ser) have six codons, and the content of ser is slightly higher than arg in the reference genome, but 10.01% of EMS mutations occurred to arg, while 7.90% occurred to ser. These results indicated the different codon and amino acid preference of EMS mutagenesis.

We next compared the frequencies of various amino acids before and after EMS mutagenesis. As shown in Supplementary Figure 5C, arg, ala, gly, and pro showed a large decrease, while asparagine (asn), isoleucine (ile), phenylalanine (phe), threonine (thr), and valine (val) showed a large increase after mutagenesis, suggesting that most of the amino acids in the former group were probably converted to the latter group by EMS mutagenesis.

### Genome-Wide “Hotspots” and “Coldspots” for EMS Mutagenesis

**FIGURE 4:**
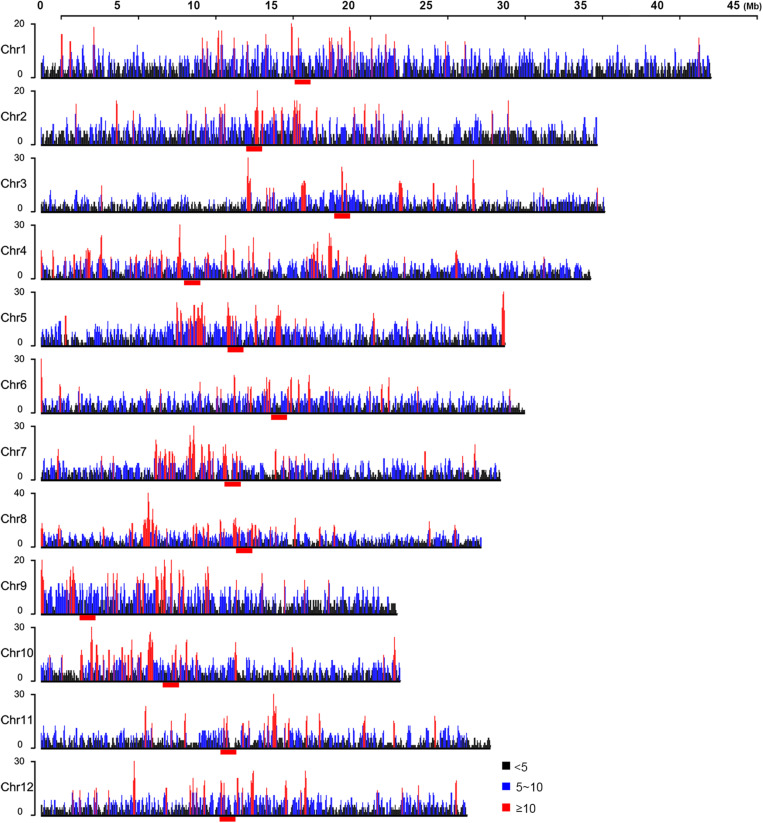
Distribution of ethyl methanesulfonate (EMS)-induced single nucleotide polymorphic sites (SNPs) along chromosomes within 100-kb bins. Black, blue, and red lines represent 100-kb bins containing <5, 5–10, and ≥10 EMS-induced SNPs, respectively. The centromeres are marked with red bars under each chromosome.

Ethyl methanesulfonate was believed to introduce mutations randomly in the genome (Koornneef et al., 1982; Kim et al., 2006). However, the 17,397 EMS-induced SNPs that we identified were apparently not uniformly distributed in the rice genome (Supplementary Figure 2B). To investigate the potential genomic factors associated with non-random distribution of EMS-induced SNPs, we divided the genome into 100-kb bins and counted the number of SNPs in each bin with a 50-kb step size, allowing approximately five SNPs in each bin and approximately two SNPs in each step on average. This yielded 535, 2,762, 3,932, and 242 bins carrying ≥10, 5–10, 0–5, and 0 EMS-induced SNPs, respectively, with an average of 4.65 SNPs in all the 100-kb bins. Based on the null hypothesis that EMS-induced SNPs were uniformly distributed, bins with SNP number ≤0.42 or ≥8.8 would be considered significantly different from the average (Chi-square test *p*-value < 0.05, df = 1). Therefore, we designated bins harboring at least 10 SNPs as “hotspots” and bins without SNPs as “coldspots” (Figure 4). Interestingly, bins harboring a high density of EMS-induced SNPs, especially the “hotspots,” tended to locate in clusters and enriched around the centromeres (Figure 4). We also conducted the sliding window analyses with 20- and 25-kb step sizes to study the distribution patterns of EMS-induced SNPs along chromosomes. Although the number of bins harboring at least 10 EMS-induced SNPs increased (from 535 to 1,092 bins for 20-kb step size and 1,353 bins for 25-kb step size), the distribution patterns were very similar with all the three step sizes (Supplementary Figure 6).

As SNPs closely linked to the causal mutation of phenotypes tended to descend together in progenies, we asked whether the bins with a higher density of EMS-induced SNPs might result from SNPs closely linked to phenotypes. The ED^6^ values were imported to estimate the correlations of SNPs with the mutant phenotypes. A region with a high density of SNPs of high ED^6^ values is potentially linked closely with the mutant phenotypes, and this was indeed proved to be true for the region harboring allelic mutations of *OsABCG3* (*LOC_Os01g61940*) (Supplementary Table 1; Chang et al., 2018). However, as shown in Supplementary Figure 7, the bins with a high density of EMS-induced SNPs on chromosomes 1, 3, 4, 6, 8, 9, 10, 11, and 12 did not show a high ED^6^ value. Some of the bins harboring SNPs with high ED^6^ value on chromosomes 2, 5, and 7 were nearby but not identical to the bins harboring a high density of EMS-induced SNPs. These results indicated that bins with a high density of SNPs were related to factor(s) other than the phenotypes.

### Genomic Factors Related to Non-random Distribution of EMS Mutagenesis

**FIGURE 5:**
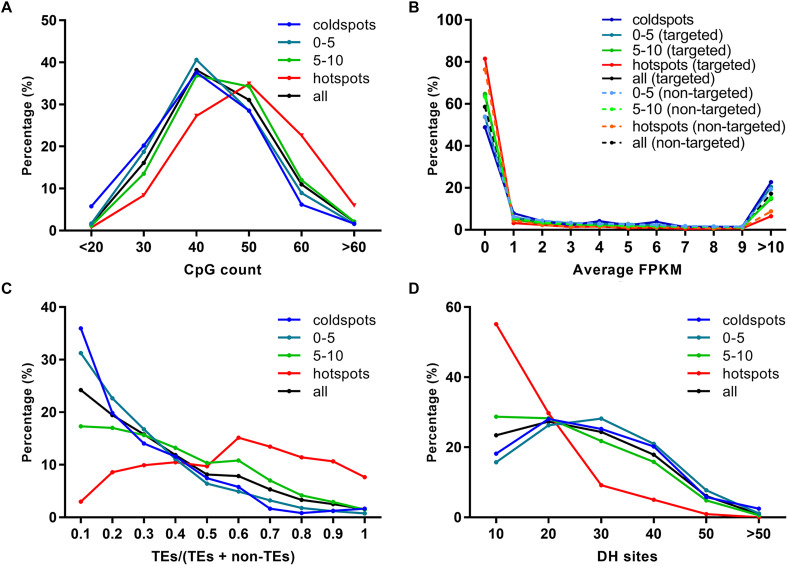
Relationship of ethyl methanesulfonate (EMS) mutagenesis with sequence context and chromatin states. **(A)** CpG count. **(B)** Average gene expression FPKM. **(C)** Ratio of transposable element (TE) genes. **(D)** Euchromatin marker DNase I hypersensitive (DH) sites. The 100-kb bins are divided into four groups according to the number of EMS-induced SNPs, including “coldspots,” 0–5, 5–10, and “hotspots.” “coldspots,” 100-kb bins without EMS-induced SNPs; 0–5, 100-kb bins with 0–5 EMS-induced SNPs; 5–10, 100-kb bins with 5–10 EMS-induced SNPs; “hotspots,” 100-kb bins with at least 10 EMS-induced SNPs; all, all the 100-kb bins in genome; targeted, genes with EMS-induced SNPs; non-targeted, genes without EMS-induced SNPs. The number of CpG, the average expression level of EMS-targeted and non-targeted genes, the ratio of TE genes, and the number of DH sites within each 100-kb bin are calculated and classified into subgroups. The percentages on the *y*-axis are then calculated as the number of subgroups divided by the total number of bins in each group.

As previously reported, EMS tended to induce SNPs at G/C sites (Greene et al., 2003; Abe et al., 2012). By analyzing the 52 HHZ mutant bulks, we found that EMS also showed a preference on sequences with relatively higher GC contents (Figures 1, 2). CpG islands, predominantly non-methylated sequences harboring more than 50% CG, are usually found in the promoters of housekeeping genes and frequently expressed genes in plants (Ashikawa, 2001; Deaton and Bird, 2011). To investigate if EMS mutagenesis has a relationship with CpG density, the CpG islands were predicted in the 5-kb sequences flanking the SNPs using Newcpgreport with default parameters (McWilliam et al., 2013). The number of CpG islands was slightly higher in the 5-kb sequences around the EMS-induced SNPs than the natural SNPs as well as the randomly selected 10,000 sites (repeated 10 times) from the reference genome (Supplementary Figure 8). Furthermore, when calculating the number of CpG islands in each bin, we found that bins with more EMS-induced SNPs were inclined to harbor more CpG islands, and the number of CpG islands was positively correlated with the number of EMS-induced SNPs within the bins (Figure 5A). About 63.55% (340) of the 535 “hotspots” harbored at least 40 CpGs, while only 36.37% (88) of the 242 “coldspots” and 44.13% (3,297) of all bins of the genome harbored at least 40 CpGs. These results indicated that EMS mutagenesis was inclined to occur in regions with high GC content and CpG density.

Chromosomes can be divided into heterochromatin and euchromatin regions according to their structures. Heterochromatin is often firmly packed and inert in transcription, while euchromatin is loosely packed with low DNA density and high transcriptional activity (Babu and Verma, 1987). TE genes are generally located in heterochromatin regions and are inert in transcription (Zhang et al., 2012). As mentioned above, the EMS-induced SNPs affected 4,726 TE genes and 6,706 non-TE genes, and most of the TE genes and 41.20% non-TE genes were expressed at low levels. We asked whether EMS mutagenesis efficiency was correlated with the expression levels of genes and the distribution of TE/non-TE genes. The average expression levels of EMS-targeted genes and non-targeted genes were estimated based on the 284 expression datasets collected from IC4R (The IC4R Project Consortium, 2016). Surprisingly, both EMS-targeted and non-targeted genes in bins harboring more EMS-induced SNPs, especially for genes located in “hotspots,” tended to express at lower levels (FPKM < 1) than genes in bins harboring fewer or no EMS-induced SNPs (Figure 5B). There were 81.54% of EMS-targeted genes and 76.34% of non-targeted genes expressed at the level of FPKM < 1 in “hotspots,” while only 48.90% of genes were expressed at the level of FPKM < 1 in “coldspots” (Figure 5B). The average FPKM of EMS-targeted genes and non-targeted genes, in “hotspots” were lower than bins harboring fewer EMS-induced SNPs (Supplementary Figure 9). The distribution of TE and non-TE genes was estimated based on the annotation of Nipponbare reference genome, and the ratio of TE/non-TE genes was calculated for each bin. As shown in Figure 5C, TE genes were overrepresented in bins with more EMS-induced SNPs, especially in EMS-induced “hotspots.” The percentages of TE genes seemed to be positively correlated with the number of EMS-induced SNPs in 100-kb bins.

DNase I hypersensitive (DH) sites have been used as symbols of regulatory elements reflecting the structure of chromatin, and regions with more DH sites tend to be euchromatin (Kodama et al., 2007; Cockerill, 2011; Zhang et al., 2012; Wang et al., 2015). It has been reported that DH sites have a strong influence on transcription activity (Kodama et al., 2007; Cockerill, 2011; Zhang et al., 2012; Wang et al., 2015). The results shown in Figures 5B,C suggest that EMS was inclined to induce SNPs in regions enriched of TE genes and less expressed genes, which are more likely to be heterochromatin. To further elucidate the relationship between EMS-induced SNPs and chromatin structures, the rice DH sites identified for seedlings and callus of Nipponbare were downloaded and compared with the Nipponbare reference genomes as described in “Materials and Methods,” resulting in 150,644 DH sites genome-wide. As expected, there were only 9.99% (746) bins enriched of TE genes (TE/non-TE > 0.5) harboring ≥5 DH sites, suggesting that the DH sites tended to be located in euchromatin regions, consistent with previous studies (Zhang et al., 2012). Only 15.14% EMS-induced “hotspots” harbored >20 DH sites, whereas 53.72% of EMS-induced “coldspots” and 49.24% of all 100-kb bins of the genome harbored >20 DH sites, suggesting that chromosome regions with a high abundance of DH sites are less favored by EMS mutagenesis (Figure 5D). Integrative comparisons of EMS-induced SNPs, CpG, TE genes, and DH sites were performed on chromosome 4 with the heterochromatin and euchromatin regions clearly defined by Zhang et al. (2012). It was clear that EMS-induced “hotspots” were more frequently located in heterochromatin regions enriched of CpG islands and TE genes but with fewer DH sites (Supplementary Figure 10). Collectively, the chromatin states and the density of CpG islands and TE genes might be associated with the non-random distribution of EMS mutagenesis in rice.

**FIGURE 6:**
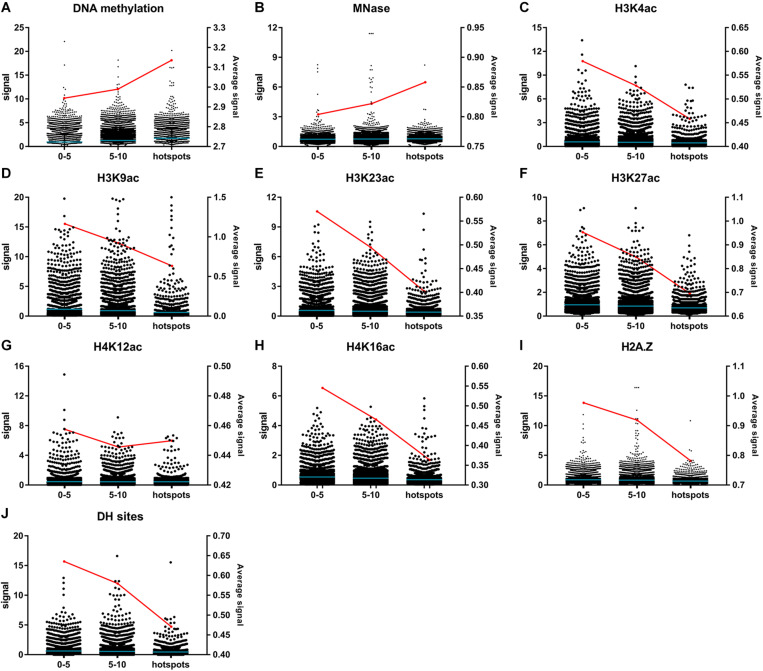
Signals of epigenetic modifications and chromatin structure-related markers around ethyl methanesulfonate (EMS)-induced single nucleotide polymorphic sites (SNPs). **(A)** DNA methylation. **(B)** MNase. **(C)** H3K4ac. **(D)** H3K9ac. **(E)** H3K23ac. **(F)** H3K27ac. **(G)** H4K12ac. **(H)** H4K16ac. **(I)** Histone variant H2A.Z. **(J)** DH sites. The signals marked with black dots (left *y*-axis) are classified according to the number of EMS-induced SNPs within 100-kb bins, with the blue lines representing the mean values. The red lines represent the average signals of each modification (right *y*-axis). The average signals are calculated with the maximum signals of SNPs located in bins harboring 0–5, 5–10, and ≥10 EMS-induced SNPs, respectively.

Epigenetic modifications, such as DNA methylation, histone methylation, histone acetylation, and histone variants, are also essential for the regulation of chromatin organization and gene expression (Liu et al., 2018). DNA methylation in plants generally occurred at CG, CHG, and CHH sites in TE genes and repeat elements to regulate the chromosome architecture and to repress the activity of TE genes (Zhang et al., 2018). Genes with DNA methylation are usually repressed in expression (Miranda and Jones, 2007; He et al., 2010; Yan et al., 2010). Micrococcal nuclease (MNase)-insensitive sites revealed by MNase-seq are usually inaccessible and protected by nucleosomes (Zhang et al., 2013; Wu et al., 2014; Mieczkowski et al., 2016). Modifications of histone, including H3K4ac (Guillemette et al., 2011; Fang et al., 2016), H3K23ac, H4K16ac (Lu et al., 2015), H3K27ac and H4K12ac (Wang et al., 2008), are usually positively correlated with gene expression levels. Nucleosomes containing histone variant H2A.Z show a preference at transcription regulatory regions and maintain chromatin accessibility (Zhang K. et al., 2017; Zhang Y. et al., 2017). To investigate whether general correlations exist between EMS mutagenesis bias and chromatin structure-related epigenetic modifications, the processed bigWig files of DNA methylation, histone acetylation, histone methylation, histone variant, and chromatin accessibility uncovered by ChIP-based sequencing methods from multiple tissues and experiments were downloaded from the PCSD database (Liu et al., 2018). To ensure the reliability of statistical analyses, only epigenetic modifications covering at least half of the EMS-induced SNPs were retained for further analyses. Considering that the genome-wide non-targeted sites were significantly more than the EMS-targeted SNPs, the average epigenetic modification signals calculated for all non-targeted sites might be reduced to a much lower level than EMS-targeted SNPs. Therefore, we compared the average signals of bins harboring different numbers of EMS-induced SNPs instead. The maximum signal of each covered SNP site and the average signals of bins harboring different numbers of SNPs were calculated for each of these epigenetic modifications as described in “Materials and Methods.” As expected, the SNPs within EMS-induced “hotspots” harbored higher signals of repressive epigenetic markers (DNA methylation and MNase) and lower signals of active epigenetic markers (H3K4ac, H3K9ac, H3K23ac, H3K27ac, H4K12ac, H4K16ac, and histone variant H2A.Z) (Figures 6A–I). The average DH site signals around EMS-induced SNPs were also negatively correlated with the number of SNPs in 100-kb bins (Figure 6J). These results suggested that the epigenetic modifications might affect the genome-wide EMS mutagenesis bias through regulation of chromatin structures.

## Discussion

Ethyl methanesulfonate is a widely used chemical alkylation mutagen in plant mutagenesis and mutation breeding of varieties with desirable phenotypes (Kim et al., 2006; Pathirana, 2011; Shoba et al., 2017). Lack of homologs of DNA alkyltransferases cannot eliminate the effects of EMS and mainly induces G/C to A/T transitions in plants (Greene et al., 2003; Kim et al., 2006; Lu et al., 2017). EMS has long been believed to introduce randomly distributed variations genome-wide, similar to physical mutagens (Koornneef et al., 1982; Greene et al., 2003; Kim et al., 2006; Sikora et al., 2011; Hola et al., 2015). Here we investigated this presumption in rice using the SNPs identified in EMS mutants.

In our previous study, we isolated 52 HHZ EMS mutants of various phenotypes. To clone the mutant genes, we deployed the SIMM method by backcrossing each of the mutants with the wild-type HHZ and then bulk-sequenced 30 F_2_ individuals of the mutant phenotype (Yan et al., 2017). The re-sequencing data of each mutant bulks were first aligned with the Nipponbare reference genome to identify the SNPs in each bulk, and then the SNPs in each of the 52 F_2_ bulks were compared among each other using the SIMM pipeline to identify the SNPs unique to each mutant bulk. These SNPs were retained as the EMS-induced SNPs for each mutant. Considering the large genome size of rice, the possibility of EMS-induced SNPs to occur at the same nucleotide in independent mutants is very low, even for those presented in highly preferred regions. Thus, exclusion of duplicated EMS-induced SNPs is also very low. This method is much more reliable than simply comparing the re-sequencing data of a single M_0_ plant and a single wild-type HHZ plant because the simple comparisons cannot distinguish the EMS-induced SNPs from the background SNPs. Besides that, the Illumina Hiseq platforms tend to produce random sequencing errors and sequencing bias that can interfere with the accurate identification of EMS-induced SNPs *via* a simple pair-wise comparison. However, by integrative comparison among the 52 mutant bulks, it is much easier to clean the background SNPs and resolve the problems caused by the Illumina Hiseq platforms. Using this method, we have identified a total of 17,397 EMS-induced SNPs in 52 mutant bulks, including the SNPs responsible for the mutant phenotypes (Chang et al., 2016a,b, 2018; Yan et al., 2017; Pan et al., 2020; Yuan et al., 2020; Zhang et al., 2020). The comparison of these SNPs with the re-sequencing data of a wild-type HHZ plant also verified the mutations. In theory, sequencing of many bulks of backcrossed F_1_ plants derived from the EMS-treated seeds can also generate the SNP information for genome-wide analysis of EMS mutagenesis features. However, as bulk-sequencing of the 52 HHZ mutants already generated a large amount of data and identified 17,397 EMS-induced SNPs, we decided to use these SNPs to analyze the genomic features associated with EMS mutagenesis.

As expected, EMS showed a preference on G/C sites, with the majority as G/C to A/T transitions. The frequency that we detected on G/C sites was 69.94%, which was very close to that of the previous report (70%) based on TILLING analysis of rice EMS mutants (Till et al., 2007). We also detected significant enrichment of dinucleotide AG at positions −1/0 and trinucleotide AAG at positions −2/−1/0 (Figure 2), suggesting that AA upstream of the mutated G, particularly A at the −1 position, provides a favorable local context for EMS mutagenesis. In *Escherichia coli*, the alkylated *O*^6^-guanidines near A/T are inclined to be removed *via* excision repair (Burns et al., 1986). However, dinucleotide AG at positions −1/0 and trinucleotides AAG and GAG at positions −2/−1/0 and AGT at positions −1/0/+1 were highly enriched in EMS mutagenesis (Figure 2), indicating possibly different DNA repair mechanisms of alkylated *O*^6^-guanidine in plants and prokaryotes.

Mutations in coding regions can alter amino acids, exon–intron splicing, transcription and translation, and even phenotypes (Chamary et al., 2006). Compared with natural SNPs presented in the Rice3K project, ∼9% more EMS-induced SNPs are located in coding regions (Figure 3). Only 22.10% EMS-induced SNPs were shared with the SNPs in the Rice3K project, and 46.38% of the shared SNPs have a MAF < 0.10 among the 3,010 rice accessions (Supplementary Table 3). For SNPs occurring in coding regions that cause NS mutations or affect exon–intron splicing, only 17.84% of them were presented in the Rice3K project. These results support the proposal that most of the EMS-induced SNPs are harmful to individuals and may be eliminated during natural propagation (Szafraniec et al., 2003). The non-synonymous SNPs induced by EMS were found to target different amino acids at different frequencies, with an apparent preference for certain codons (Supplementary Figure 5).

To investigate the genome-wide distribution patterns of EMS-induced SNPs, the 17,397 EMS-induced SNPs that we detected in HHZ were classified into different groups according to the number of SNPs in 100-kb bins with a 50-kb step size. We found that bins harboring dense EMS-induced SNPs were irrelevant to the mutant phenotypes and often clustered on chromosomes, especially the centromere regions (Figure 4 and Supplementary Figure 7). We also conducted the sliding window analyses with 20- and 25-kb step sizes and found all three step sizes to result in very similar distribution patterns (Supplementary Figure 6). Centromeres, the chromosomal regions that are often highly packed, are important for eukaryotic cell division and replication. Repeat elements including satellite tandem repeats and transposable elements are enriched in centromeres, and genes within centromeres are often expressed at low levels (Zhong et al., 2002). Compared with “coldspots” lacking EMS-induced SNPs, we found that bins carrying more EMS-induced SNPs, especially the “hotspots,” were inclined to harbor higher contents of TE genes and less DH sites (Figure 5), suggesting that the chromatin structure might play a role in regulating EMS mutagenesis.

DNA methylation and post-translational histone modification can alter chromatin state and affect gene expression (van Leeuwen and van Steensel, 2005; He et al., 2011; Zhang et al., 2018). We deducted that more genes located in EMS-induced “hotspots” tended to be expressed at lower levels (Figure 5B). Consistently, the average signals for the repressive markers (DNA methylation and MNase insensitive sites) were higher for SNPs in “hotspots” than in bins with fewer EMS-induced SNPs (Figure 6). On the contrary, the average signals for the active epigenetic markers (histone acetylation and histone variant H2A.Z) were lower for SNPs in “hotspots” (Figure 6). The more repressive epigenetic modifications and less active epigenetic modifications might increase the interactions between EMS and chromatin or hinder the repair of mutations induced by EMS (Burns et al., 1986), resulting in the EMS mutagenesis bias in heterochromatin regions with higher contents of GC and TE genes and less transcription.

Methylation at histone 3 lysine 4 (H3K4me) is another active epigenetic marker, and it can regulate H3K4ac at active gene promoters (Guillemette et al., 2011). Genes with H3K36me1 at transcription start sites (TSS) are generally repressed, while genes with H3K36me3 at both TSS and gene body are expressed at high levels (Hu et al., 2020). H3K27me3 is a repression hallmark of chromatin, and genes with H3K27me3 tend to be repressed (Barski et al., 2007; Deal and Henikoff, 2011). These epigenetic markers were excluded from our analysis due to the low coverage of EMS-induced SNPs. With more data of these epigenetic modifications becoming available, we can further address the relationship between chromatin structure and EMS mutagenesis bias more clearly.

In conclusion, we demonstrated a non-random distribution of EMS-induced SNPs in rice in this study. The method for identification of EMS-induced SNPs could be applied to other plant species to evaluate if EMS mutagenesis bias is pervasive. Integrative analysis of local sequence context, gene expression data, and chromatin structure-related epigenetic modifications not only provides a roadmap for exploring possible mechanisms involved in EMS mutagenesis bias and DNA repair but also helps in the selection of target genes with desirable phenotypes and determination of a population size for mutant screening of target genes in mutation breeding.

## Conclusion

Ethyl methanesulfonate-induced mutations provide numerous sources for screening individuals with desirable traits in plants. EMS was believed to introduce variations randomly in genome. However, multiple allelic EMS-induced mutants were often identified during mutant screening, indicating a potential bias of EMS mutagenesis. By integrative analysis of the re-sequencing data, gene expression data, and epigenetic modification data, we found a preference of EMS mutagenesis in sequences with a relatively higher GC content in heterochromatin. The chromatin structure and epigenetic modifications on chromatin might also contribute to the EMS mutagenesis bias. These findings not only verify the bias of EMS mutagenesis but also indicate the potential roles of epigenetic modifications and chromatin structure in EMS mutagenesis and DNA repair.

## Data Availability Statement

The re-sequencing data from this article are deposited in the NCBI Sequence Read Archive under accession number PRJNA283022.

## Author Contributions

WY conducted the data analysis. WY and XT designed the experiments and wrote the article. XWD and CY reviewed the article. All authors contributed to the article and approved the submitted version.

## Conflict of Interest

The authors declare that the research was conducted in the absence of any commercial or financial relationships that could be construed as a potential conflict of interest.

## Footnotes

1 https://mvd.iaea.org/

2 http://www.ncbi.nlm.nih.gov/geo/

## Supplementary Material

Supplementary Figure 1 Statistics of ethyl methanesulfonate (EMS)-induced single nucleotide polymorphic sites (SNPs) in each mutant bulk. The *y*-axis indicates the number of EMS-induced SNPs in each mutant bulk. The dashed line represents the average number of EMS-induced SNPs among all mutant bulks. The SNPs in each mutant bulk are separated into nine groups according to their SNP index (SI) from 0.2 to 1 and indicated by different colors in each bar. SNPs with SI < 0.1 are excluded due to low reliability. SNPs closely linked to the mutant phenotypes have a SI close to 1, whereas unlinked SNPs have a SI close to 0.5.

Supplementary Figure 2 Statistics of distances between adjacent ethyl methanesulfonate (EMS)-induced single nucleotide polymorphic sites (SNPs). **(A)** Average distance between adjacent EMS-induced SNPs in each mutant bulk. **(B)** Statistics of all EMS-induced SNPs based on distances between adjacent SNPs. All the 17,397 SNPs are fixed on the rice genome for measurement of distances between adjacent SNPs. The SNPs are then divided into different groups based on the distance between adjacent SNPs as shown in the *x*-axis, and the number of SNPs in each group was counted and shown in the *y*-axis. The average distance for all EMS-induced SNPs is 21.43 kb.

Supplementary Figure 3 Distribution of percentages of each dinucleotide and trinucleotide between ethyl methanesulfonate (EMS)-induced single nucleotide polymorphic sites (SNPs) and natural SNPs. **(A)** Dinucleotides at positions −1/0. **(B)** Dinucleotides at positions 0/+1. **(C)** Trinucleotides at positions −2/−1/0. **(D)** Trinucleotides at positions −1/0/+1. **(E)** Trinucleotides at positions 0/+1/+2. The red and blue dots are the clearly enriched dinucleotides and trinucleotides identified in EMS-induced SNPs with *p*-values < 0.05 and ≥0.05, respectively. The black dots are the remaining dinucleotides and trinucleotides with no big differences between EMS-induced SNPs and natural SNPs. The red and black dashed lines represent the expected and observed linear regression line in each comparison, respectively. The observed correlation *R*^2^ is shown at the top.

Supplementary Figure 4 Statistics of average GC contents in 50-bp flanking sequences around EMS-induced SNPs, natural SNPs, and randomly selected sites. The dashed line represents the average GC contents across the 10 random selections of ∼10,000 sites based on the Nipponbare reference genome.

Supplementary Figure 5 Statistics of amino acid conversions induced by ethyl methanesulfonate (EMS). **(A)** EMS-induced single nucleotide polymorphic sites (SNPs) varied the codons at different frequencies. The red dashed line represents the percentages of each codon in the Nipponbare reference genome. **(B)** EMS-induced SNPs varied the amino acids at different frequencies. The red line and the blue line represent the percentages of EMS-targeted amino acids and their corresponding percentages in the Nipponbare reference genome, respectively. **(C)** Varied percentage of each amino acid by EMS.

Supplementary Figure 6 Distribution of ethyl methanesulfonate (EMS)-induced single nucleotide polymorphic sites (SNPs) along chromosomes in 100-kb bins with 20-kb step size. The number of EMS-induced SNPs in each overlapped 100-kb bin was calculated. The black, blue, and red lines represent 100-kb bins containing <5, 5–10, and ≥10 EMS-induced SNPs, respectively. The centromeres are marked with red bars under each chromosome.

Supplementary Figure 7 Distribution of ethyl methanesulfonate (EMS)-induced single nucleotide polymorphic sites (SNPs) and ED^6^ values along chromosomes. The EMS-induced SNPs are counted in overlapped 100-kb bins with 50-kb step size. The ED are calculated and raised to the power of 6 for each EMS-induced SNP as described in “Materials and Methods.” In the **upper panel**, the black, blue, and red bars represent 100-kb bins carrying <5, 5–10, and ≥10 EMS-induced SNPs, respectively. The **lower panel** indicates the ED^6^ values of each EMS-induced SNP (black, ED < 0.8; blue, 0.8 < ED < 1.2; red, ED ≥ 1.2). A higher ED^6^ value indicates stronger linkage with mutant phenotype.

Supplementary Figure 8 Statistics of the number of CpG islands within 5-kb sequences flanking the ethyl methanesulfonate (EMS)-induced single nucleotide polymorphic sites (SNPs) and natural SNPs and simulated SNPs. The number of CpG islands in each 5-kb flanking sequence was determined with Newcpgreport software and indicated in the *x*-axis. All the 5-kb flanking sequences were classified into different groups according to the number of CpG islands that they carried. Then, the number of 5-kb sequences in each group was added up and divided by the total number of EMS-induced SNPs and natural SNPs, giving the percentages on the *y*-axis. The black arrow indicates the crossing point where the percentage related to EMS-induced SNPs changed from lower to higher in comparison with the natural SNPs and simulated SNPs. The simulated SNPs are randomly selected from the reference genome and repeated 10 times (dashed line with error bars, *N* = 10); 10,000 loci per simulation.

Supplementary Figure 9 Average expression levels of ethyl methanesulfonate (EMS)-targeted genes and non-targeted genes. The average fragments per kilobase of transcript per million mapped reads (FPKM) values were calculated for the EMS-targeted genes and non-targeted genes in “coldspots,” “hotspots,” and bins harboring 0–5 and 5–10 SNPs, respectively.

Supplementary Figure 10 Distribution of ethyl methanesulfonate (EMS)-induced single nucleotide polymorphic sites (SNPs) and CpG, transposable element (TE) genes, and DNase I hypersensitive (DH) sites on chromosome 4. The centromere is marked with the horizontal red bar. The 100-kb bins with low (SNPs < 5, CpG < 20, TE < 30%, DH < 20), median (SNPs < 10, CpG < 40, TE < 60%, DH < 40), and high (SNPs ≥ 10, CpG ≥ 40, TE ≥ 60%, DH ≥ 40) number of EMS-induced SNPs, CpG, TE genes, and DH sites are marked in black, blue, and red, respectively. The digitally straightened pachytene chromosome 4 stained with DAPI at the bottom is modified from Zhang et al. (2012). The heterochromatin and euchromatin regions are shown as dark and gray, respectively. The euchromatin–heterochromatin boundary on the long arm is marked by black lines.

Supplementary Table 1 List of the identified genome-wide 17,397 ethyl methanesulfonate-induced single nucleotide polymorphic sites.

Supplementary Table 2 Statistics of ethyl methanesulfonate-induced single nucleotide polymorphic sites in the 52 Huanghuazhan mutant bulks.

Supplementary Table 3 List of ethyl methanesulfonate-induced single nucleotide polymorphic sites presented in the Rice3K project.
