# Supplementary figures and images for: The Genome-Wide EMS Mutagenesis Bias Correlates With Sequence Context and Chromatin Structure in Rice
Source: Front Plant Sci. 2021 Mar 24;12:579675. doi: 10.3389/fpls.2021.579675 (PMC8025102; doi:10.3389/fpls.2021.579675)

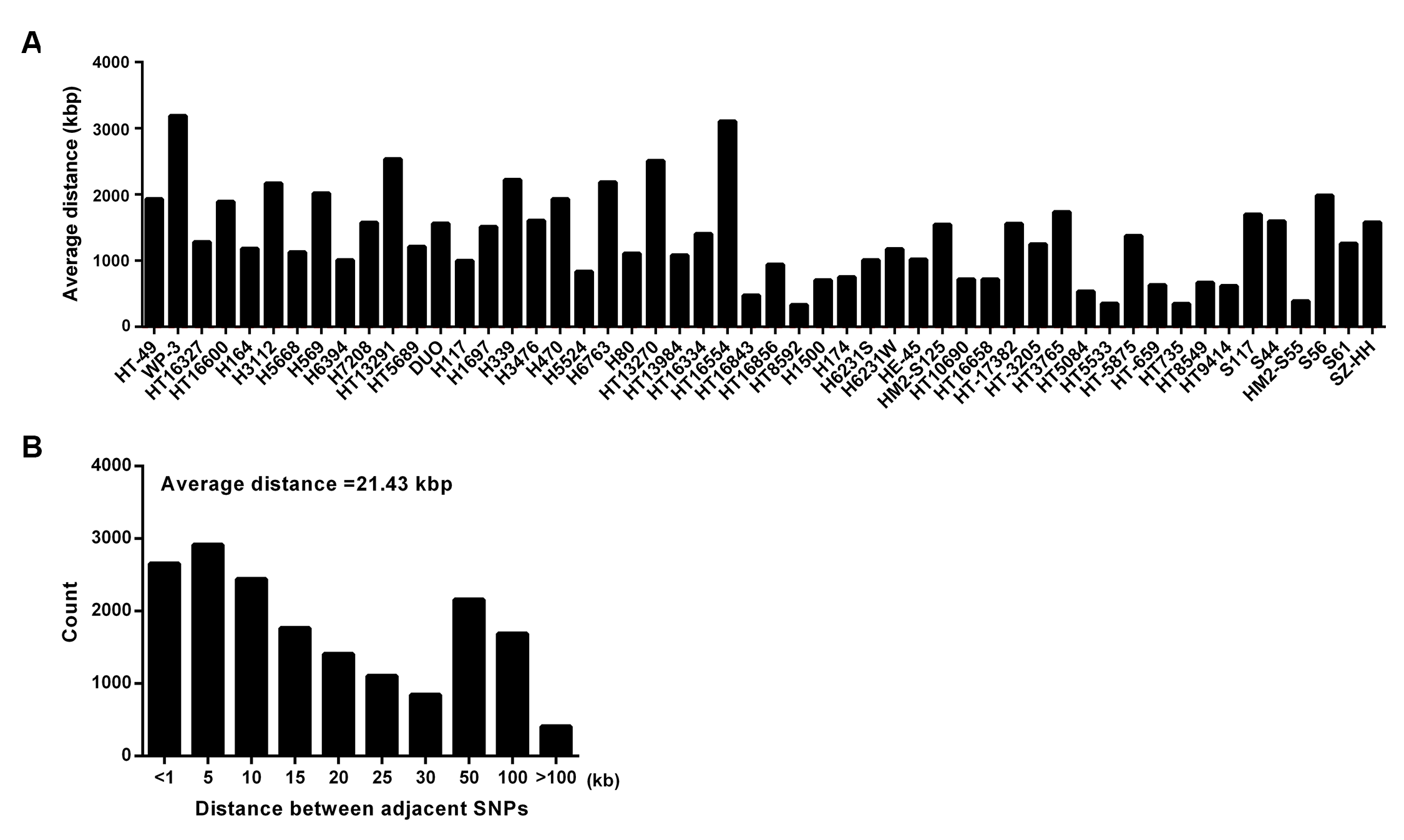

Supplement: Supplementary Figure 2 — Statistics of distances between adjacent ethyl methanesulfonate (EMS)-induced single nucleotide polymorphic sites (SNPs). (A) Average distance between adjacent EMS-induced SNPs in each mutant bulk. (B) Statistics of all EMS-induced SNPs based on distances between adjacent SNPs. All the 17,397 SNPs are fixed on the rice genome for measurement of distances between adjacent SNPs. The SNPs are then divided into different groups based on the distance between adjacent SNPs as shown in the x-axis, and the number of SNPs in each group was counted and shown in the y-axis. The average distance for all EMS-induced SNPs is 21.43 kb. [file Image_2.TIFF]

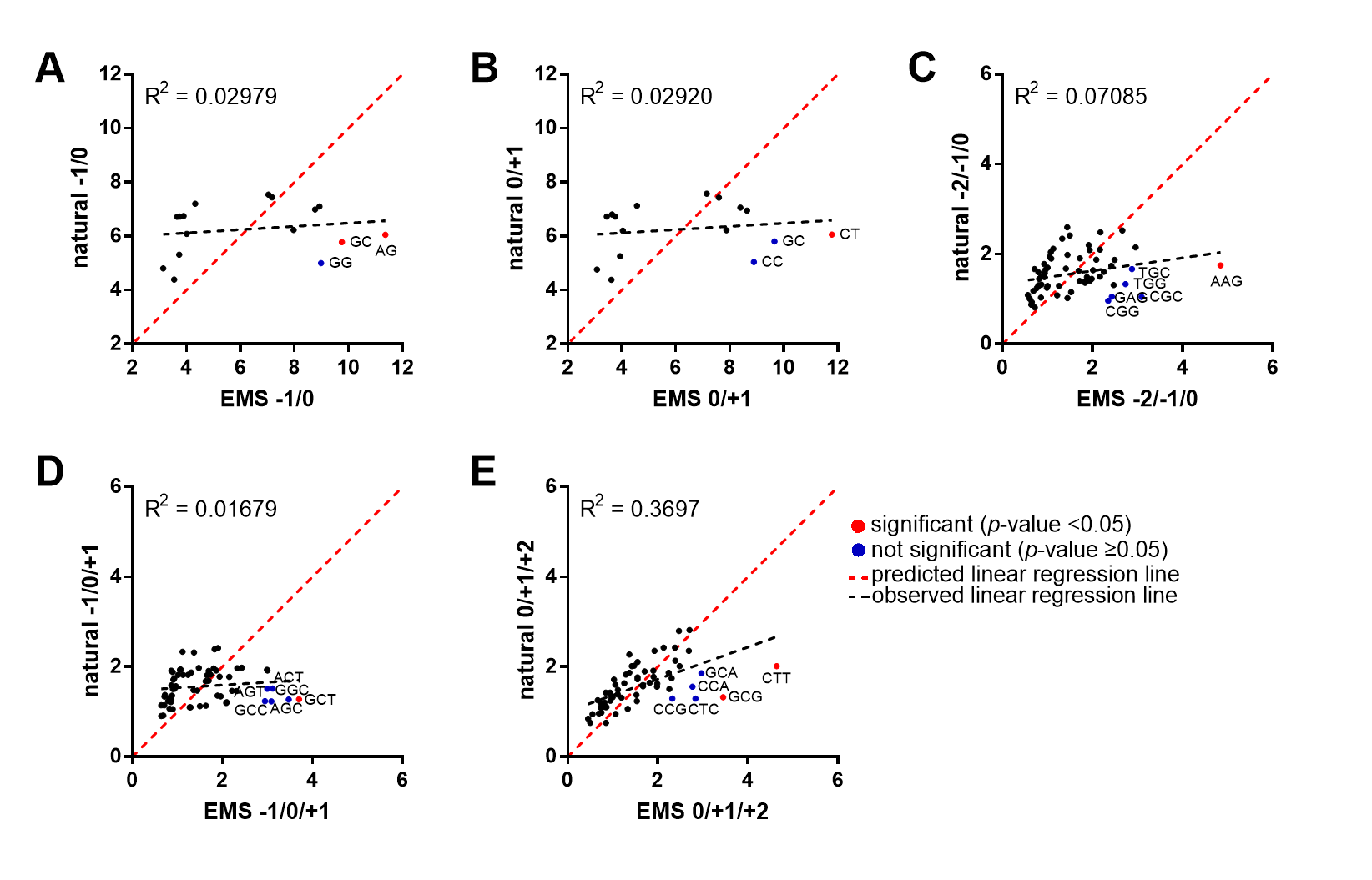

Supplement: Supplementary Figure 3 — Distribution of percentages of each dinucleotide and trinucleotide between ethyl methanesulfonate (EMS)-induced single nucleotide polymorphic sites (SNPs) and natural SNPs. (A) Dinucleotides at positions −1/0. (B) Dinucleotides at positions 0/+1. (C) Trinucleotides at positions −2/−1/0. (D) Trinucleotides at positions −1/0/+1. (E) Trinucleotides at positions 0/+1/+2. The red and blue dots are the clearly enriched dinucleotides and trinucleotides identified in EMS-induced SNPs with p-values < 0.05 and ≥0.05, respectively. The black dots are the remaining dinucleotides and trinucleotides with no big differences between EMS-induced SNPs and natural SNPs. The red and black dashed lines represent the expected and observed linear regression line in each comparison, respectively. The observed correlation R2 is shown at the top. [file Image_3.TIF]

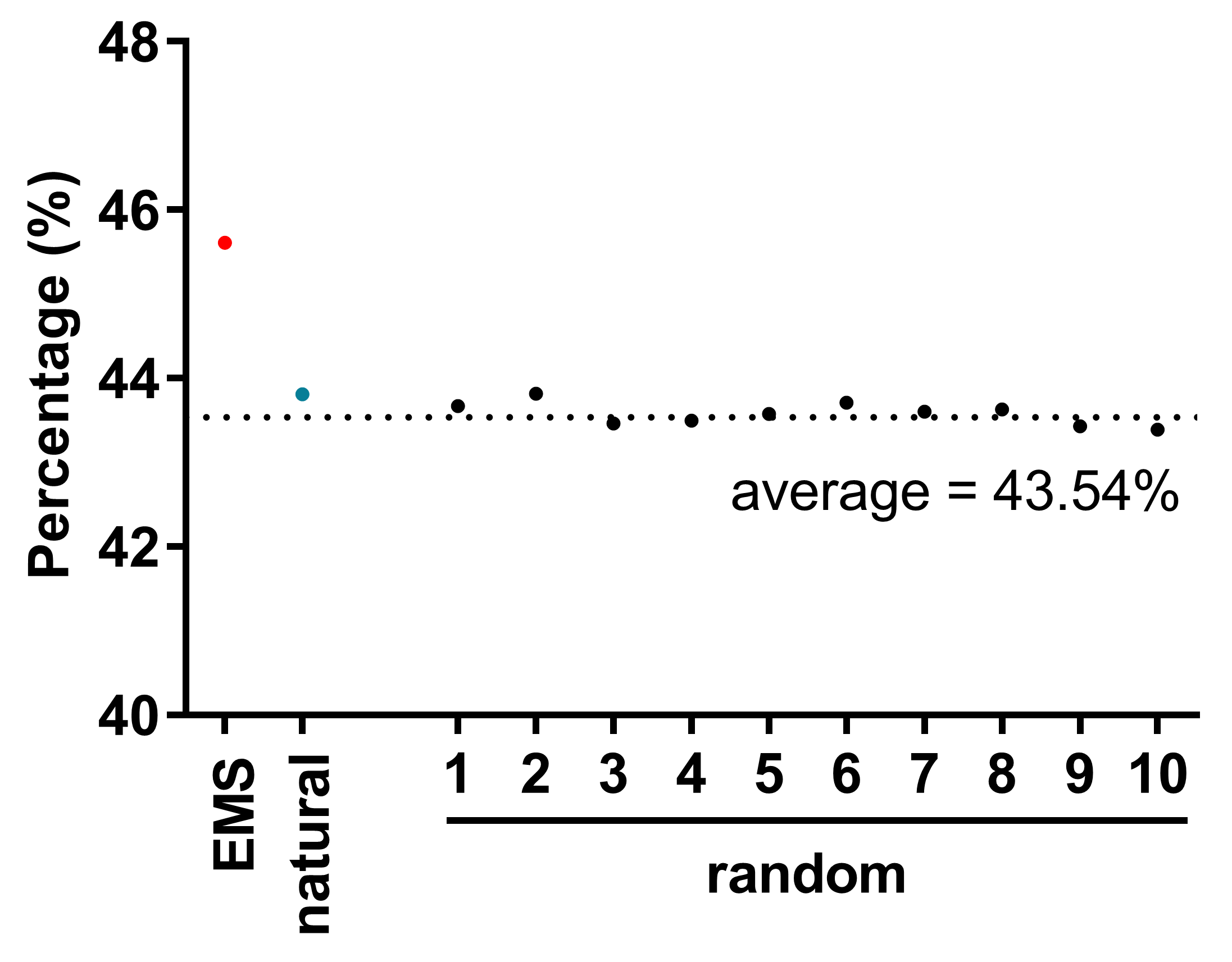

Supplement: Supplementary Figure 4 — Statistics of average GC contents in 50-bp flanking sequences around EMS-induced SNPs, natural SNPs, and randomly selected sites. The dashed line represents the average GC contents across the 10 random selections of ∼10,000 sites based on the Nipponbare reference genome. [file Image_4.TIF]

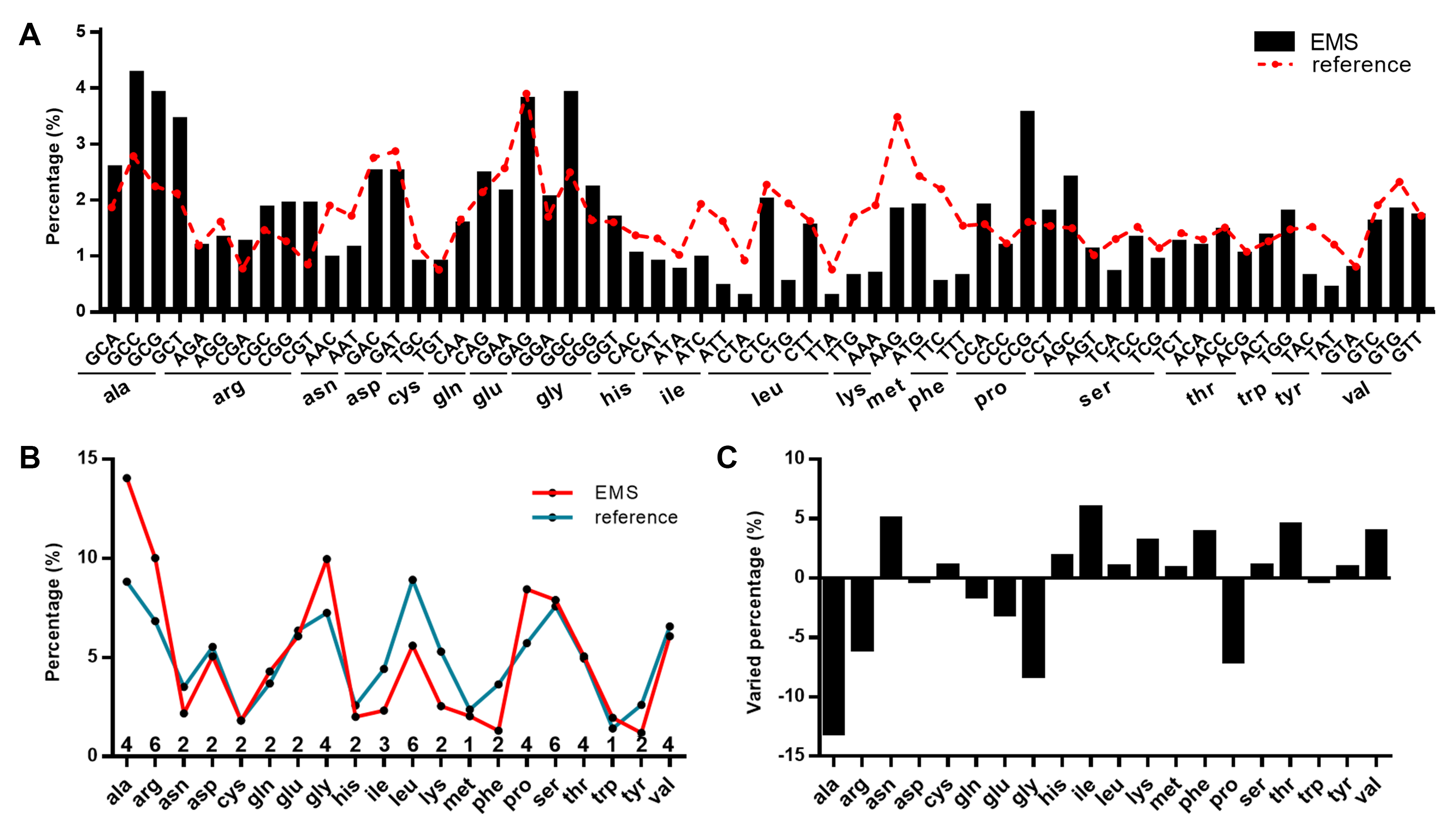

Supplement: Supplementary Figure 5 — Statistics of amino acid conversions induced by ethyl methanesulfonate (EMS). (A) EMS-induced single nucleotide polymorphic sites (SNPs) varied the codons at different frequencies. The red dashed line represents the percentages of each codon in the Nipponbare reference genome. (B) EMS-induced SNPs varied the amino acids at different frequencies. The red line and the blue line represent the percentages of EMS-targeted amino acids and their corresponding percentages in the Nipponbare reference genome, respectively. (C) Varied percentage of each amino acid by EMS. [file Image_5.TIF]

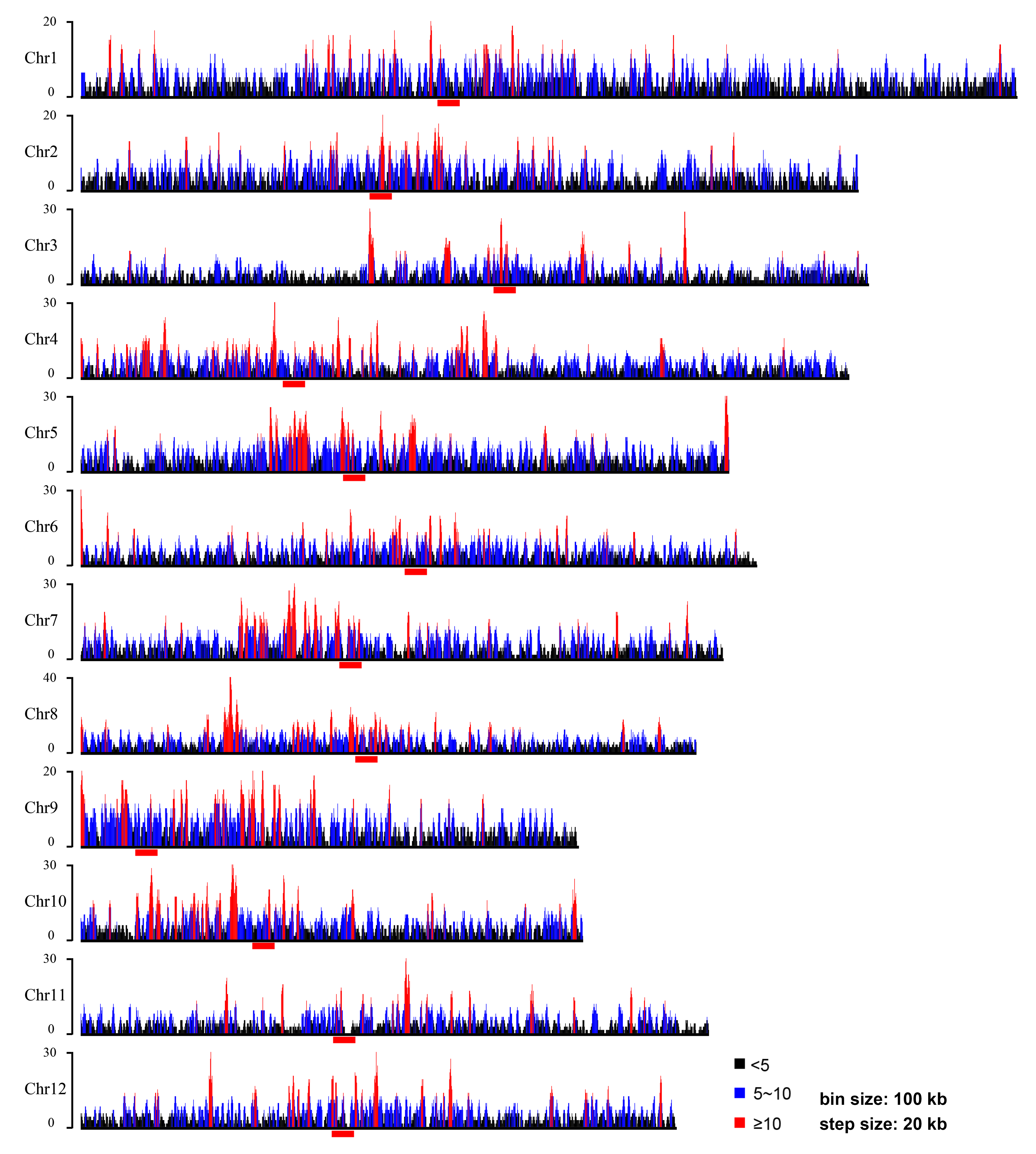

Supplement: Supplementary Figure 6 — Distribution of ethyl methanesulfonate (EMS)-induced single nucleotide polymorphic sites (SNPs) along chromosomes in 100-kb bins with 20-kb step size. The number of EMS-induced SNPs in each overlapped 100-kb bin was calculated. The black, blue, and red lines represent 100-kb bins containing <5, 5–10, and ≥10 EMS-induced SNPs, respectively. The centromeres are marked with red bars under each chromosome. [file Image_6.TIF]

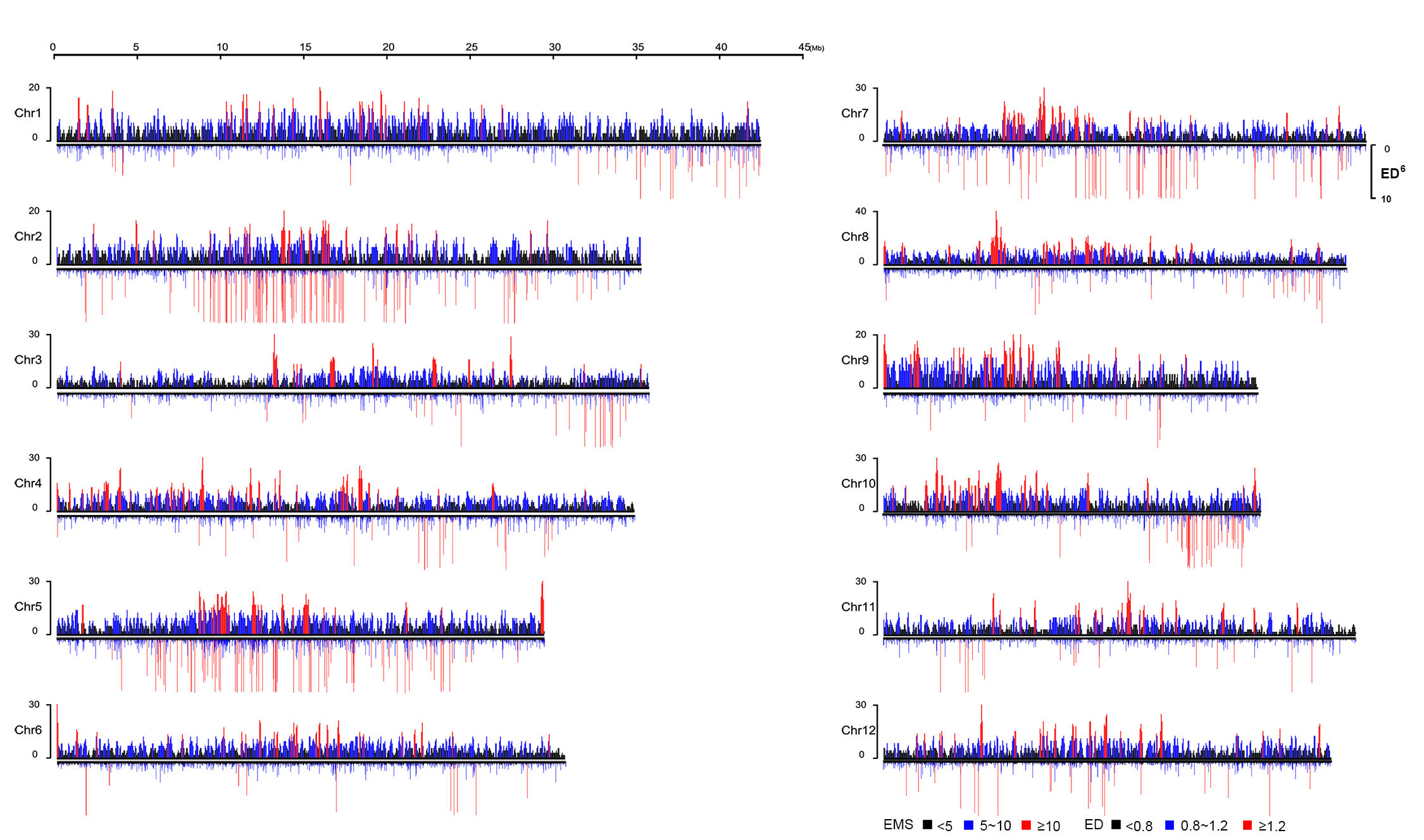

Supplement: Supplementary Figure 7 — Distribution of ethyl methanesulfonate (EMS)-induced single nucleotide polymorphic sites (SNPs) and ED6 values along chromosomes. The EMS-induced SNPs are counted in overlapped 100-kb bins with 50-kb step size. The ED are calculated and raised to the power of 6 for each EMS-induced SNP as described in “Materials and Methods.” In the upper panel, the black, blue, and red bars represent 100-kb bins carrying <5, 5–10, and ≥10 EMS-induced SNPs, respectively. The lower panel indicates the ED6 values of each EMS-induced SNP (black, ED < 0.8; blue, 0.8 < ED < 1.2; red, ED ≥ 1.2). A higher ED6 value indicates stronger linkage with mutant phenotype. [file Image_7.TIFF]

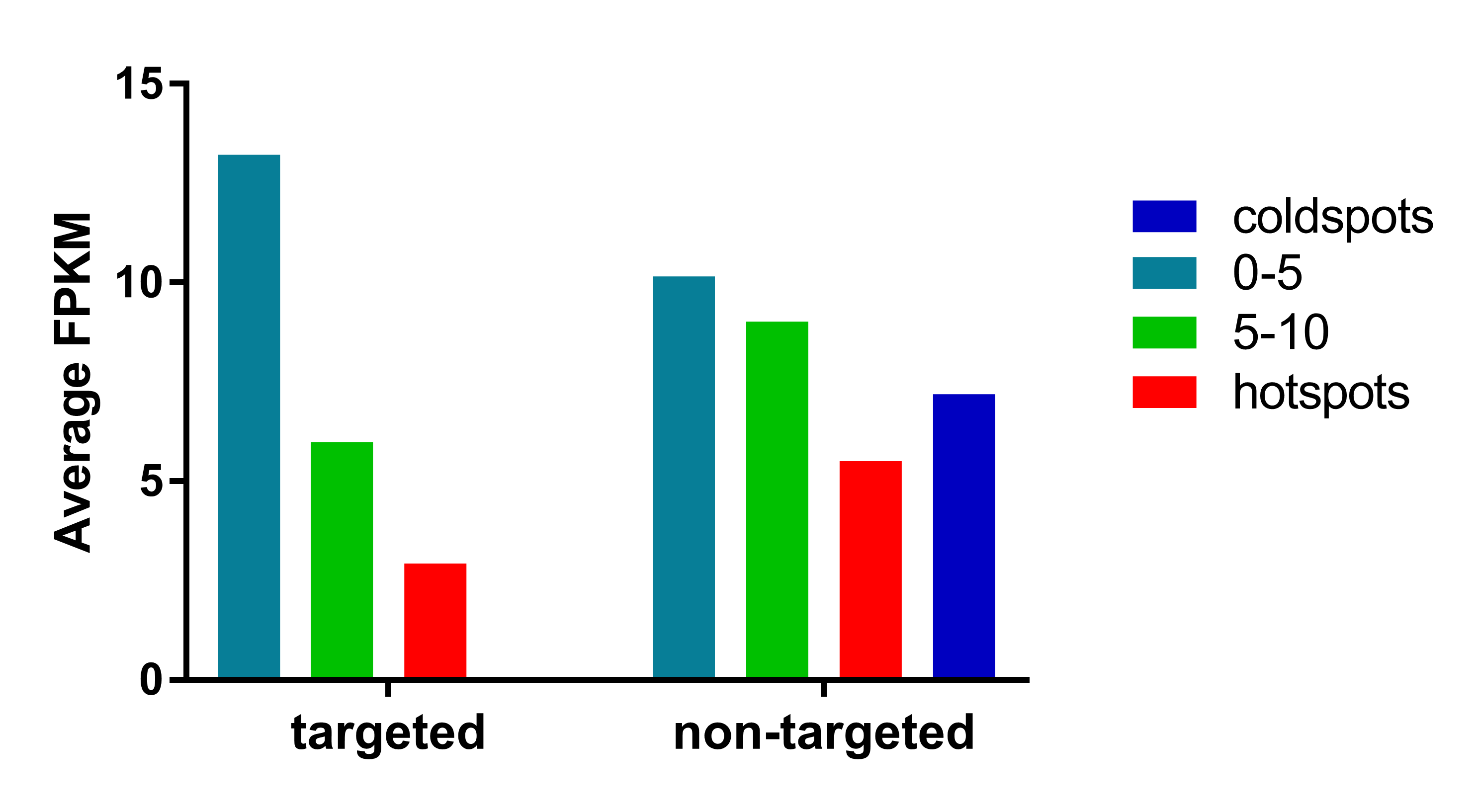

Supplement: Supplementary Figure 9 — Average expression levels of ethyl methanesulfonate (EMS)-targeted genes and non-targeted genes. The average fragments per kilobase of transcript per million mapped reads (FPKM) values were calculated for the EMS-targeted genes and non-targeted genes in “coldspots,” “hotspots,” and bins harboring 0–5 and 5–10 SNPs, respectively. [file Image_9.TIF]

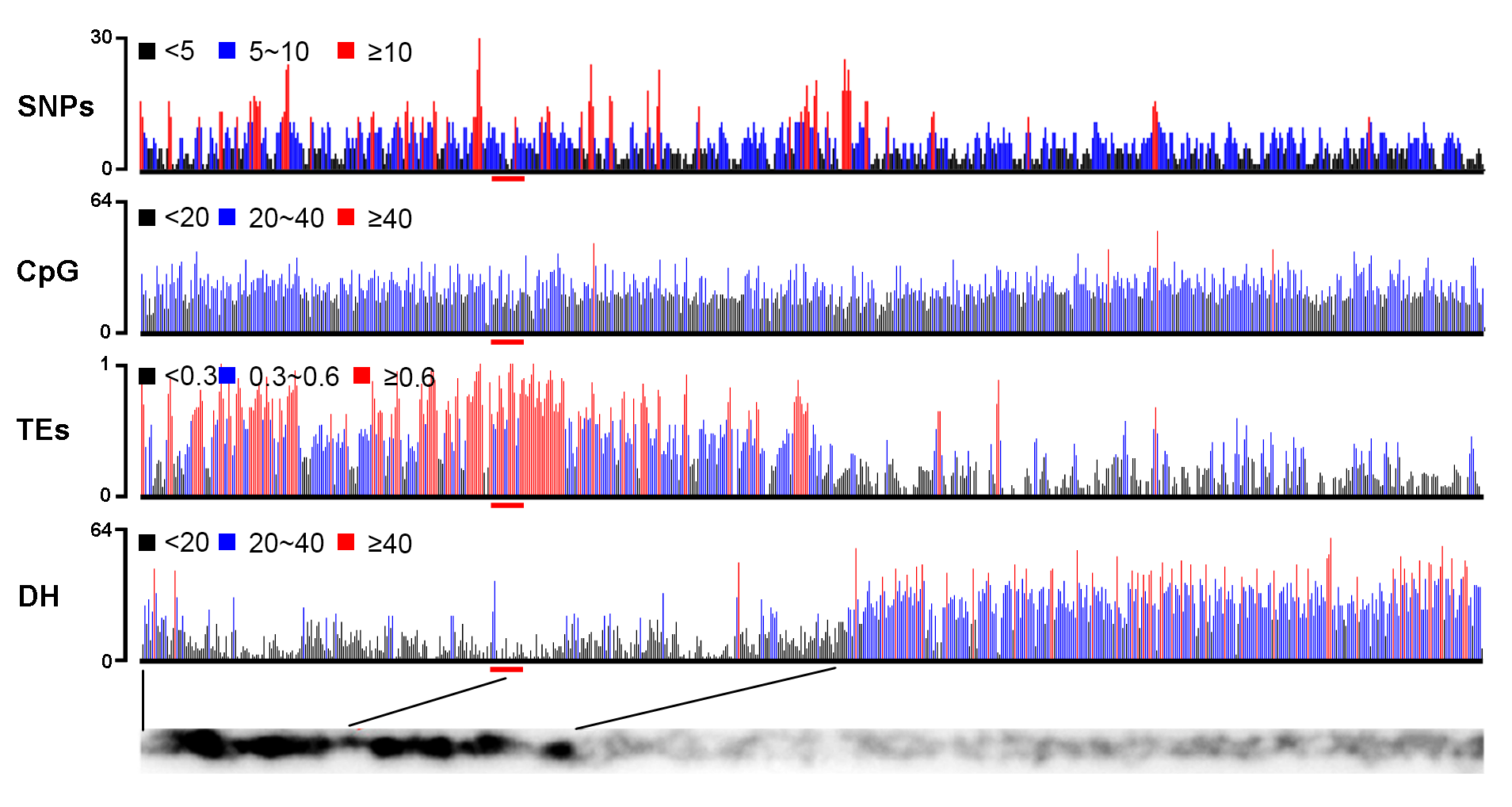

Supplement: Supplementary Figure 10 — Distribution of ethyl methanesulfonate (EMS)-induced single nucleotide polymorphic sites (SNPs) and CpG, transposable element (TE) genes, and DNase I hypersensitive (DH) sites on chromosome 4. The centromere is marked with the horizontal red bar. The 100-kb bins with low (SNPs < 5, CpG < 20, TE < 30%, DH < 20), median (SNPs < 10, CpG < 40, TE < 60%, DH < 40), and high (SNPs ≥ 10, CpG ≥ 40, TE ≥ 60%, DH ≥ 40) number of EMS-induced SNPs, CpG, TE genes, and DH sites are marked in black, blue, and red, respectively. The digitally straightened pachytene chromosome 4 stained with DAPI at the bottom is modified from Zhang et al. (2012). The heterochromatin and euchromatin regions are shown as dark and gray, respectively. The euchromatin–heterochromatin boundary on the long arm is marked by black lines. [file Image_10.TIFF]
